# Simultaneous Occurrence of Berlin Nodule and Optic Disc Granuloma: A Rare Case Report

**DOI:** 10.22336/rjo.2026.59

**Published:** 2026

**Authors:** Nidhi Kalra, Jaspreet Singh Makkar, Mohini Agrawal

**Affiliations:** 1 Department of Cornea and Anterior Segment, Command Hospital, Chandimandir, Punjab, India; 2 Department of Ophthalmology, Command Hospital, Chandimandir, Punjab, India; 3 Department of Ophthalmology, Command Hospital, Udhampur, India

**Keywords:** ocular sarcoidosis, Berlin nodule, iris granuloma, optic nerve head granuloma, multimodal imaging, ACE = Angiotensin-Converting Enzyme, AC = Anterior Chamber, BCVA = Best-Corrected Visual Acuity, CT = Computed Tomography, HRCT = High-Resolution Computed Tomography, IOP = Intraocular Pressure, IWOS = International Workshop on Ocular Sarcoidosis, MRI = Magnetic Resonance Imaging, OCT = Optical Coherence Tomography, SD-OCT = Spectral-Domain Optical Coherence Tomography, ONH = Optic Nerve Head, PAS = Peripheral Anterior Synechia, RNFL = Retinal Nerve Fibre Layer, TB = Tuberculosis, UBM = Ultrasound Biomicroscopy, VF = Visual Field

## Abstract

**Purpose:**

To describe a rare case of sarcoidosis presenting with simultaneous anterior segment granuloma (Berlin nodule) and posterior (optic nerve head) granuloma.

**Methods:**

A 40-year-old male presented with a four-year history of persistent glare in the right eye. Detailed ocular examination, gonioscopy, ultrasound biomicroscopy (UBM), spectral-domain optical coherence tomography (SD-OCT), laboratory evaluation, chest computed tomography (CT), endobronchial lymph node biopsy, and magnetic resonance imaging (MRI) of the brain and orbits were performed.

**Results:**

The right eye revealed mild granulomatous anterior uveitis, a yellowish-white iris nodule at 6 o’clock, mild vitritis, optic disc granuloma obscuring the cup, and peripheral vitreous snowballs. The left eye showed mild anterior uveitis with peripheral vitreous snowballs. UBM confirmed the Berlin nodule as a hyporeflective trabecular lesion, and SD-OCT demonstrated increased temporal peripapillary thickness. Gonioscopy documented localized angle involvement without significant synechial closure. Laboratory investigations revealed elevated serum angiotensin-converting enzyme, with negative Mantoux and Quantiferon-TB Gold tests. CT chest demonstrated bilateral hilar lymphadenopathy with ground-glass opacities, and endobronchial biopsy confirmed non-caseating granulomatous lymphadenitis, establishing systemic sarcoidosis. MRIs of the brain and orbits were normal, excluding neurosarcoidosis. The patient was treated with oral and topical corticosteroids, resulting in complete regression of both granulomas.

**Discussion:**

This case expands the clinical spectrum of ocular sarcoidosis by demonstrating the exceptionally rare coexistence of a Berlin nodule and an optic nerve head granuloma. Recognition of these highly specific granulomatous lesions, supported by multimodal imaging and systemic evaluation, facilitated prompt diagnosis, avoided unnecessary invasive intervention, and resulted in complete resolution with corticosteroid therapy while preserving vision.

**Conclusion:**

This case highlights the exceedingly rare coexistence of Berlin nodule and optic nerve head granuloma as presenting features of systemic sarcoidosis. Early recognition, multimodal imaging, and histopathology are critical for accurate diagnosis and timely management.

## Introduction

Sarcoidosis is a chronic, multisystem granulomatous disorder of uncertain etiology, characterized by non-caseating granulomas that most commonly involve the lungs and lymph nodes, but can also affect the eyes, skin, heart, and nervous system [1,2]. Ocular involvement occurs in 20-40% of patients and may serve as the first clue to systemic disease [1]. Uveitis is the most common manifestation, usually granulomatous and bilateral, whereas other ocular features, such as conjunctival granulomas, lacrimal gland involvement, episcleritis, scleritis, and neuro-ophthalmic sarcoidosis, are less frequent.

Optic nerve involvement is reported in 5-15% of sarcoidosis cases, typically presenting as optic neuritis, disc edema, or atrophy [3,4]. Primary optic disc granulomas are rare and can mimic intraocular tumors [4]. Similarly, anterior segment nodules, such as Koeppe at the pupillary margin, Busacca within the iris stroma, and Berlin nodules in the trabecular meshwork, are uncommon but represent highly specific ocular signs [1].

The concurrent presence of an iris nodule and an optic disc granuloma in the same patient is exceptionally unusual. We present such a case in a 40-year-old male, in which early recognition, supported by IWOS criteria, multimodal imaging, and histopathology, enabled timely management.

## Case presentation

A 40-year-old male presented with persistent glare in the right eye for four years. He denied redness, pain, photophobia, or floaters. Best-corrected visual acuity (BCVA) was 6/9 in the right eye, improving to 6/6 and 6/6 in the left eye. Slit-lamp evaluation revealed few granulomatous keratic precipitates, anterior chamber cells 2+, and a yellowish-white 2×2 mm iris nodule at 6 o’clock in the anterior chamber (**Fig. 1A, B**). Fundus showed grade 1 vitritis, a nodular granuloma at the temporal optic disc margin obliterating the cup, and few vitreous snowballs. Left eye showed medium-sized keratic precipitates and 0.5+ anterior chamber cells. The fundus had grade 1 media haze, a normal disc with a 0.4 cup-to-disc ratio, and peripheral vitreous snowballs. Intraocular pressures were normal in both eyes. No choroidal granulomas, vasculitis, or retinitis were noted.

**Fig. 1:**
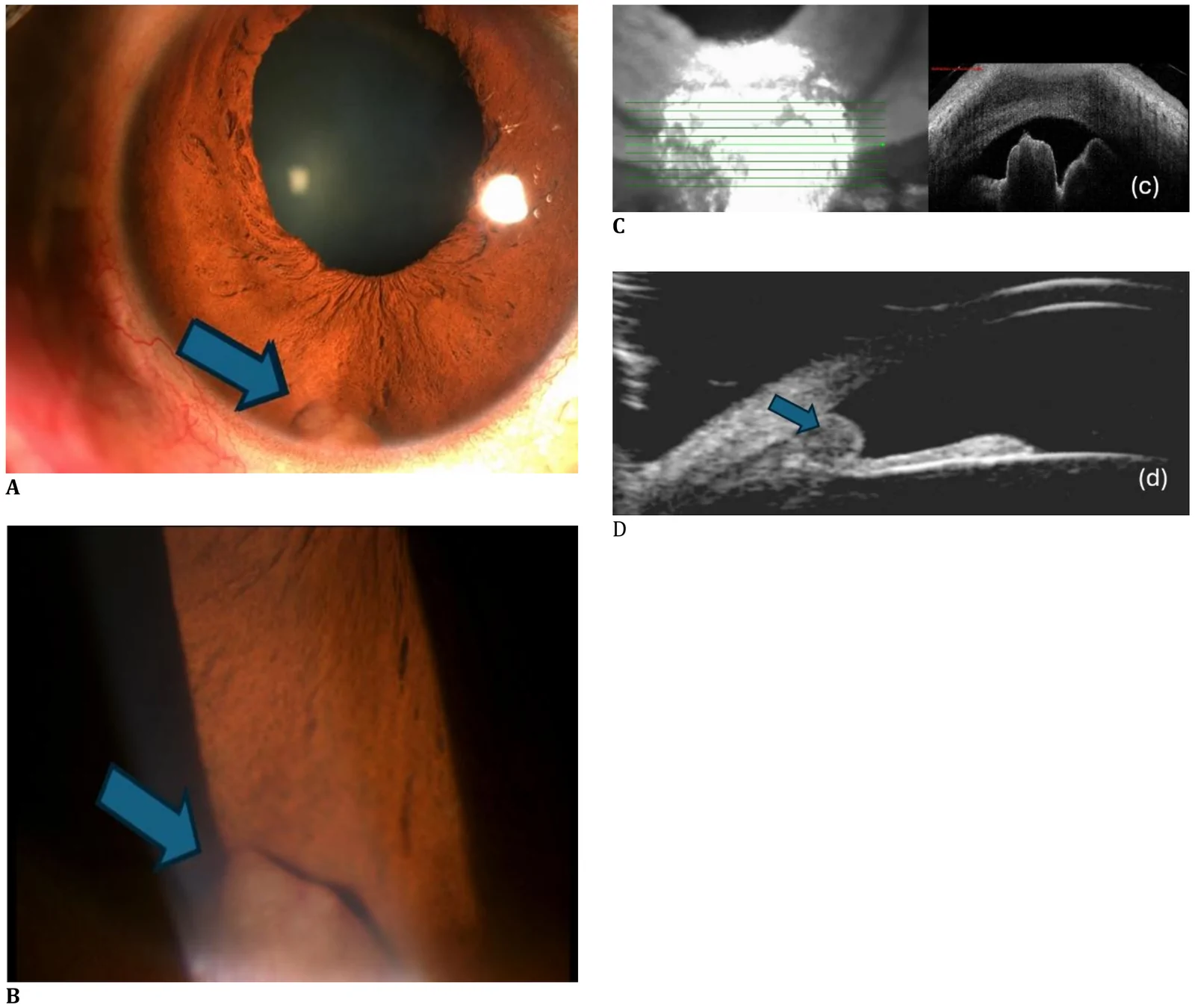
Baseline anterior segment of the affected eye. **A**. Diffuse-illumination slit-lamp photograph showing a solitary, yellowish-white iris nodule at the 6 o’clock position in the anterior chamber; **B**. High-magnification (x40) slit-lamp view highlighting the pearly-white surface with fine feeder vessels and crisp borders; **C**. Anterior-segment optical coherence tomography (AS-OCT) demonstrating a hyperreflective nodular lesion arising from the iris with posterior shadowing; **D**. Ultrasound biomicroscopy (UBM; 50-MHz probe) showing a well-defined mass arising from the anterior chamber angle/iris root

Baseline gonioscopy revealed open angles with Shaffer grade III–IV in the right eye and grade II–III in the left eye, with a focal trabecular meshwork nodule corresponding to the Berlin nodule at 6 o’clock. **Anterior segment OCT confirmed dome-shaped hyporeflective nodules with hyperreflective borders (Fig. 1C). Ultrasound biomicroscopy showed Berlin nodules as hyporeflective angle-based lesions (Fig. 1D)**. Spectral-domain-OCT revealed localized temporal peripapillary retinal nerve fibre layer thickening corresponding to optic disc granulomas (**Fig. 2A-C**).

**Fig. 2:**
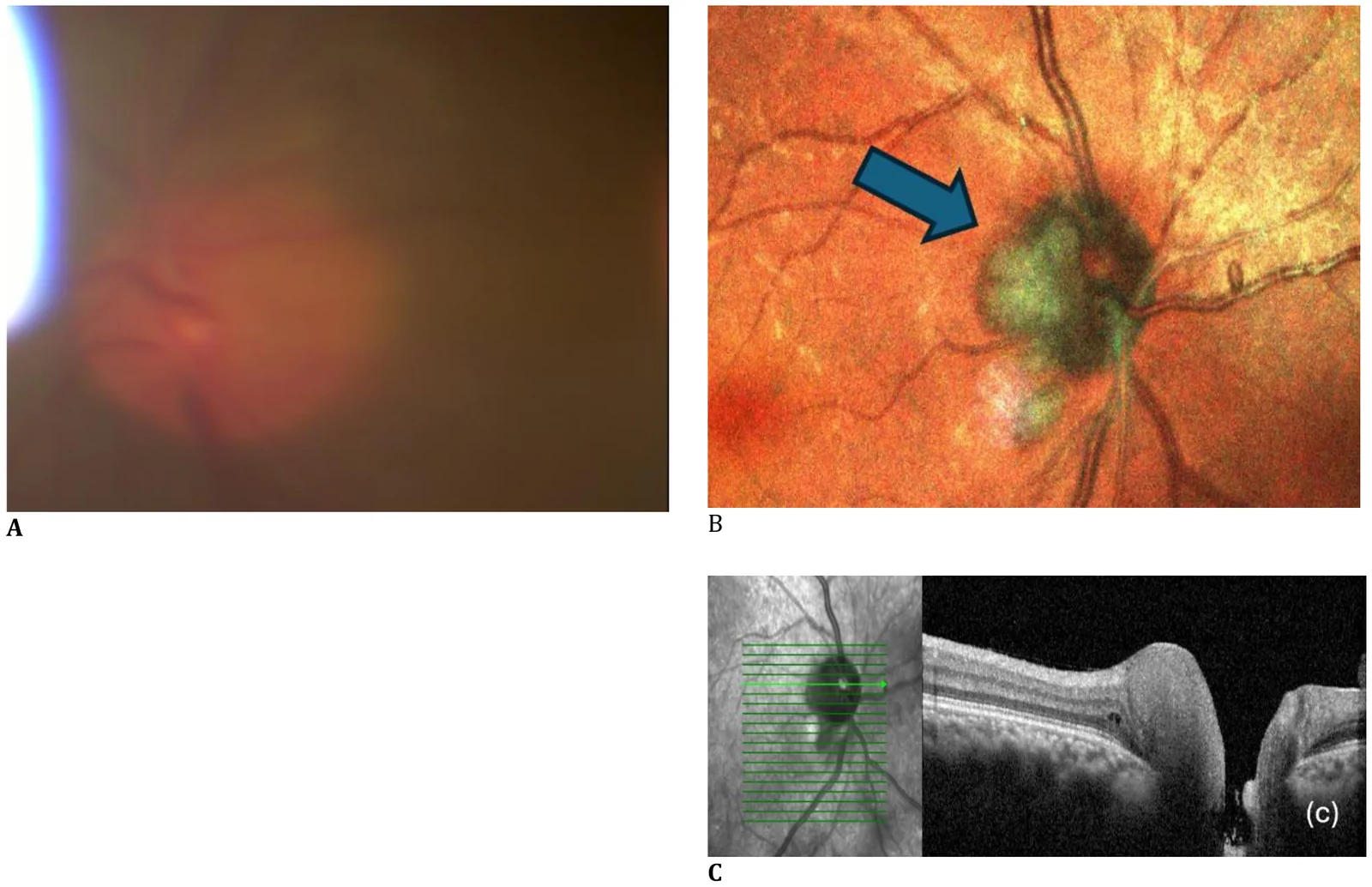
Posterior segment at presentation (right eye). **A**. 90-diopter biomicroscopic photograph showing blurred temporal disc margins with an irregular, elevated mass over the temporal half of the optic disc, obscuring the physiologic cup, features suggestive of an optic disc granuloma; **B**. Multicolor pseudocolor image (Spectralis) delineating the irregular disc-associated mass over the temporal neuroretinal rim; **C**. Spectral-domain OCT (SD-OCT; Spectralis) horizontal peripapillary scan demonstrating increased temporal peripapillary thickness corresponding to the clinically visible lesion

Routine laboratory investigations were unremarkable. Mantoux test and Quantiferon-TB Gold test were negative, and serologies for syphilis and HIV were also normal. Serum angiotensin-converting enzyme (ACE) was elevated (79.8 U/L; normal 12-68 U/L). HRCT of the chest revealed bilateral hilar lymphadenopathy with ground-glass opacities in the lungs (**Fig. 3A**). Endobronchial lymph node biopsy confirmed non-caseating epithelioid cell granulomas composed of histiocytes and giant cells, establishing the diagnosis of systemic sarcoidosis (**Fig. 3B**). MRI of the brain and orbits was normal.

**Fig. 3:**
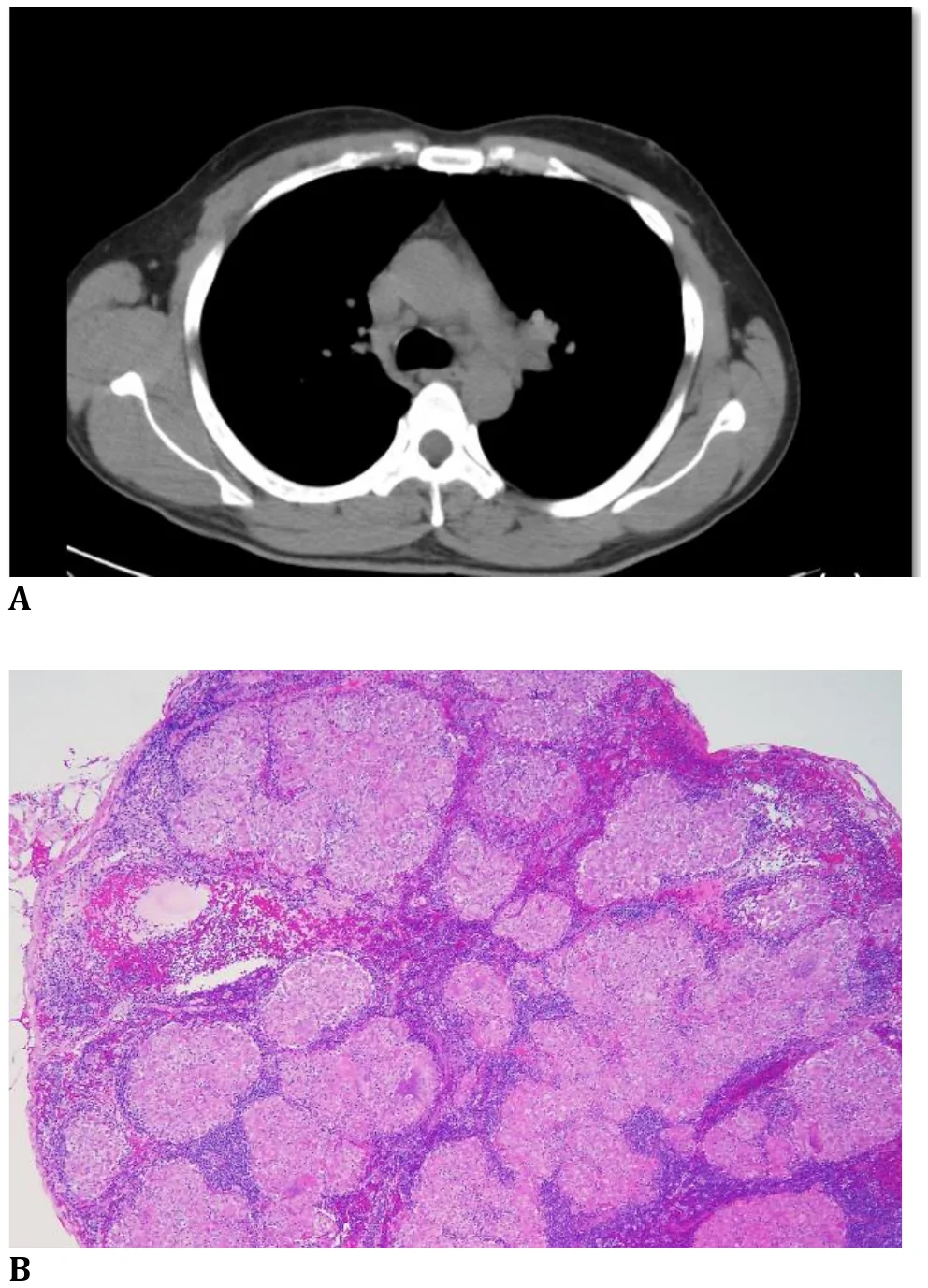
Systemic work-up supporting granulomatous disease. **A**. Non-contrast computed tomography of the chest depicting enlarged hilar lymph nodes, concordant with granulomatous lymphadenopathy; **B**. Histopathological examination (hematoxylin-eosin stain) of a lymph-node biopsy showing multiple discrete and confluent non-necrotizing epithelioid granulomas

The patient was treated with oral prednisolone (1 mg/kg/day) and topical prednisolone acetate 1% applied four times daily, gradually tapered. At one-month follow-up, both the iris nodule and optic disc granuloma showed regression (**Fig. 4A-E**). A residual peripheral anterior synechia (PAS) involving approximately 1 clock hour at the 6 o’clock position was observed (**Fig. 4B**). IOP remained stable during follow-up. Repeat SD-OCT demonstrated resolution of peripapillary thickening (**Fig. 4E**). 

**Fig. 4:**
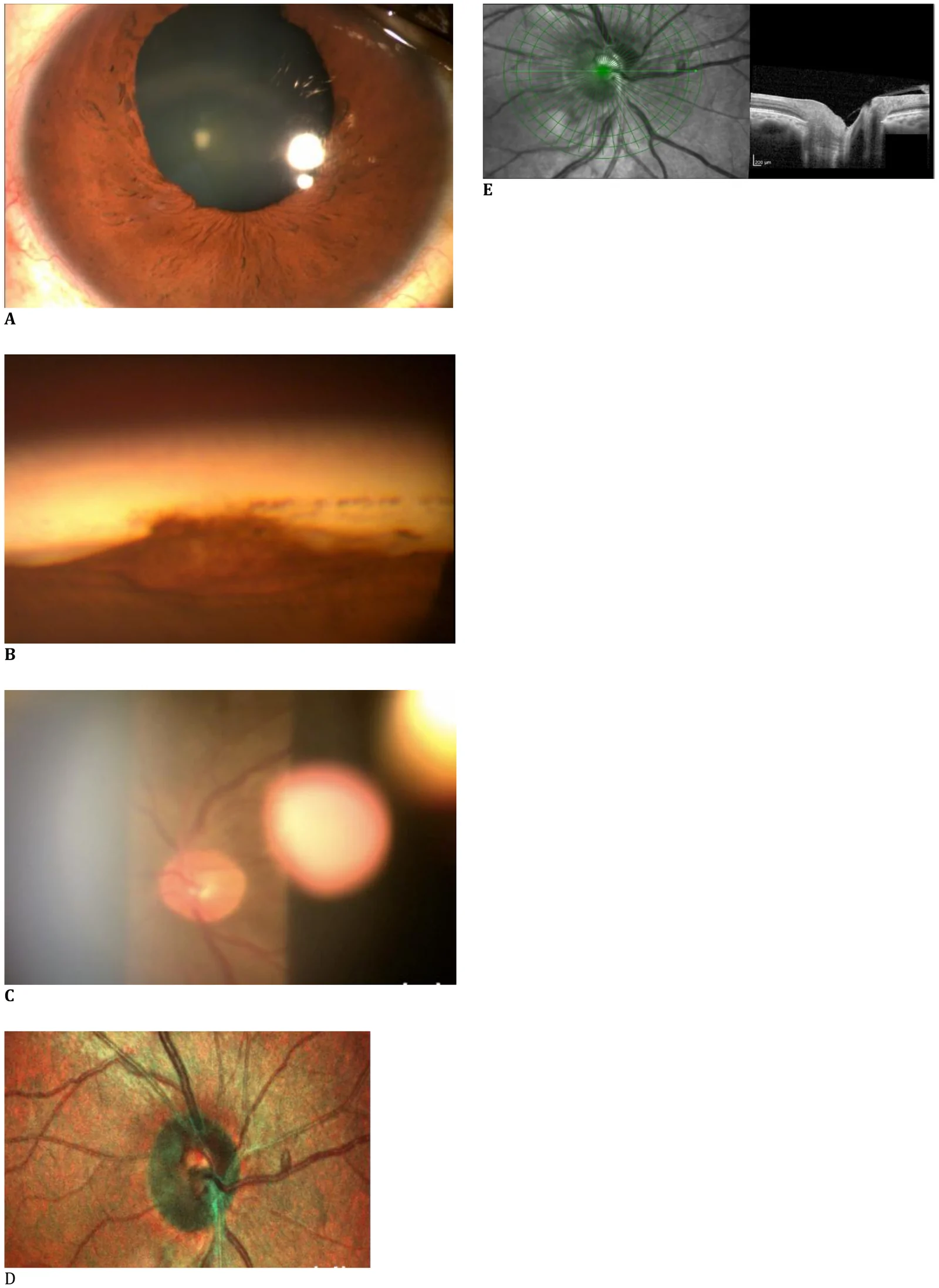
Follow-up after therapy. **A**. Slit-lamp photograph showing complete resolution of the iris nodule at the 6 o’clock position; **B**. Gonioscopic view demonstrating peripheral anterior synechiae at the prior lesion site with no residual angle mass; **C**. 90-diopter biomicroscopic image of the right optic disc with sharp, well-defined margins and resolution of the optic disc granuloma, the optic cup is re-visualized; **D**. Multicolor pseudocolor image confirming restoration of normal disc contour and margins; **E**. SD-OCT (Spectralis) showing reduction of temporal peripapillary thickening toward baseline, consistent with structural resolution

## Discussion

Ocular sarcoidosis is diagnostically challenging due to its diverse presentations and overlap with infectious and neoplastic disorders [1-4]. Nodular granulomas, whether iris, trabecular, or optic disc, are highly specific but rare signs. The concurrent presence of an iris nodule and optic disc granuloma, as in our patient, is exceptionally uncommon and broadens the recognized spectrum of ocular sarcoidosis.

Berlin nodules are rare trabecular meshwork granulomas, first detailed by Hamanaka et al., and may obstruct aqueous outflow, causing secondary glaucoma [5-8]. Our patient exhibited Berlin nodules that resolved with corticosteroid therapy, reinforcing their value as early indicators of sarcoidosis. Moreover, only a limited PAS (1 clock hour) persisted after resolution of the active lesion, and IOP remained stable throughout the follow-up period. Nevertheless, such cases warrant long-term glaucoma follow-up, as sarcoid-related angle granulomas and synechiae can predispose to secondary glaucoma. Regular gonioscopy, optic nerve head evaluation, and visual field testing should therefore be incorporated into the monitoring plan.

Prior reports have described optic nerve head granulomas mimicking intraocular tumors and, in some cases, leading to unnecessary enucleation [9]. In contrast, our patient’s lesion was recognized early and regressed with corticosteroids, preventing unnecessary radical intervention.

Optic nerve head granulomas in sarcoidosis may fall within the neurosarcoidosis spectrum; hence, neuroimaging is recommended to exclude additional intracranial or meningeal disease. In our patient, MRIs of the brain and orbits were unremarkable, thereby localizing the granulomatous involvement to the optic nerve head without evidence of systemic neurosarcoidosis.

Although ocular sarcoidosis frequently involves both anterior and posterior segment structures, the **simultaneous presence of Berlin nodules and optic nerve head granuloma** in the same patient is exceedingly rare. Most descriptions of Berlin nodules detail isolated anterior uveitis manifestations, without concurrent ONH involvement [6-8]. A few case reports have documented isolated optic disc granulomas or anterior segment granulomas in sarcoidosis [9,10], but concurrent granulomas of the anterior angle and optic nerve head, as observed in our case, have seldom been reported.

According to the Revised International Workshop on Ocular Sarcoidosis (2019), our patient demonstrated multiple ocular signs, a trabecular meshwork nodule, optic disc granuloma, vitreous snowballs, and bilateral granulomatous uveitis, alongside systemic evidence of elevated ACE, radiological hilar lymphadenopathy, and histopathological confirmation [10]. Together, these satisfied the criteria for a definitive diagnosis of ocular sarcoidosis.

This case is remarkable for its rare dual granulomatous presentation in a middle-aged male. Our patient was diagnosed early, treated appropriately, and achieved complete resolution with preserved vision. The case highlights the importance of multimodal ophthalmic imaging and systemic evaluation in avoiding misdiagnosis and guiding therapy.

## Conclusion

This case highlights the rare coexistence of Berlin nodules and optic nerve head granuloma as the manifestation of systemic sarcoidosis. Comprehensive multimodal imaging, systematic use of IWOS criteria, histopathologic confirmation, and exclusion of mimickers are crucial for diagnosis. Prompt corticosteroid therapy led to rapid regression of both lesions, while residual PAS underscores the importance of long-term glaucoma surveillance. The addition of brain and orbital MRIs, which were normal, further excluded neurosarcoidosis. Our report emphasizes that meticulous ocular and systemic evaluation is essential for guiding management and preventing vision-threatening sequelae in sarcoid uveitis.
